# Psychosocial working conditions and chronic low-grade inflammation in geriatric care professionals: A cross-sectional study

**DOI:** 10.1371/journal.pone.0274202

**Published:** 2022-09-15

**Authors:** Helena C. Kaltenegger, Matthias Weigl, Linda Becker, Nicolas Rohleder, Dennis Nowak, Caroline Quartucci

**Affiliations:** 1 Institute and Clinic for Occupational, Social and Environmental Medicine, University Hospital, LMU Munich, Munich, Germany; 2 Institute for Patient Safety, University Hospital Bonn, Bonn, Germany; 3 Chair of Health Psychology, Institute of Psychology, Friedrich-Alexander University Erlangen-Nürnberg, Erlangen, Germany; 4 Bavarian Health and Food Safety Authority, Institute for Occupational Health and Product Safety, Environmental Health, Munich, Germany; Texas State University, UNITED STATES

## Abstract

**Background:**

Chronic low-grade inflammation has been suggested as a key factor in the association between stress exposure and long-term health. Care work is recognized as a profession with a high degree of job stress and health risks. However, for care professionals, the study base on inflammatory activity due to adverse working conditions is limited.

**Objective:**

The aim of this study was to explore associations between self-reported psychosocial working conditions and care professionals’ biomarkers of systemic low-grade inflammation.

**Methods:**

*N* = 140 geriatric care professionals (79.3% females, mean age = 44.1 years) of six care facilities were enrolled in a cross-sectional study consisting of standardized medical examinations and employee surveys. Standardized questionnaires were used for evaluation of psychosocial work characteristics (work overload, job autonomy, social support) based on Karasek’s job strain model. Blood samples were drawn for two biomarkers of inflammatory activity: C-reactive protein (CRP) and leukocyte count. Analyses comprised uni- and multivariate logistic and linear regression analyses.

**Results:**

We determined a proportion of 5.4% of care professionals with increased low-grade inflammation. We further observed a relationship between job autonomy and CRP, such that reports of high job autonomy were associated with increased levels of CRP (adjusted OR = 4.10, 95% CI [1.10, 15.26], *p* = .035), which was robust in additional analyses on further potential confounders. No significant associations with participants’ leukocyte numbers were found.

**Conclusions:**

This exploratory study contributes to the research base on links between workplace stress and ensuing illness in care professionals. Our findings may help to identify risk and protective factors of the work environment for chronic low-grade inflammation. The results require further scrutiny, and future prospective studies on associations of psychosocial working conditions, low-grade inflammation and long-term health outcomes in care professionals are needed.

## Introduction

Care work is associated with a substantial level of job stress involving major risks to psychophysiological health [1–3]. Chronic exposure to work stressors has been linked to physical and mental morbidity, including cardiovascular diseases (CVD), type 2 diabetes, clinical depression, etc., as well as mortality [4–8].

An increasing research base indicates that systemic low-grade inflammation as a sub-component of the immune system plays a critical role in the development of chronic conditions [9–12]. Systemic inflammatory markers, such as C-reactive protein (CRP) or circulating leukocytes, are suggested to be involved in the atherosclerotic process [13, 14]. Specifically for exposure to workplace stress, previous investigations revealed relationships between adverse and unfavorable working conditions and chronic systemic low-grade inflammation among employees [15–19]. Persistent workplace stress has been found to impact immune function with higher likelihoods of increased inflammatory activity and reduced adaptive immune function in employees reporting poor psychosocial working conditions [20–22]. This resonates well with existing knowledge and evidence stemming from psychoneuroimmunology that experiences of acute and chronic psychosocial stress affect human immune function and inflammatory processes [9, 23, 24].

Despite its growing importance, the respective research base on work stress, immune function, and inflammatory processes specifically for care professionals is limited and inconsistent [25–27]. Previous investigations into work-related influences on nursing professionals’ immune system predominantly surveyed the role of shift work [28, 29], overall professional stress [30, 31], and mediating experiences of job satisfaction [32]. Given the eminent role of psychosocial risk factors and their contribution to long-term health outcomes, protuberant knowledge gaps remain.

First, available studies on the associations of nurses’ job stress with immunological and inflammatory biomarkers suggest that high work stress environments affect both cellular and humoral immunity [30, 31, 33–35]. Most studies however, used aggregate measures of job stress and focused on specific branches of the immune system (such as cellular immunity or immunoglobulins). To our knowledge, studies considering different components of psychosocial work stress are scarce [33, 34]. Investigations that take into account different job characteristics, including stressors as well as resources, and their individual as well as interactive effects provide a better understanding of how work stress affects care professionals’ immune system.

Second, concerning the role of protective factors at work, a range of psychosocial resources have been scrutinized with particular emphasis on autonomy and social support [2, 36, 37]. However, the current research base remains inconclusive with some hints (yet, outside of nursing) suggesting that high social support and job control are associated with lower inflammation status [18, 19, 38–41].

Third, work stress models have proven useful in understanding the pathways from workplace stress to altered immune and inflammation processes [12]. Among these, one of the most prominent approaches is the job demand-control model (JDC; [42, 43]) and expanded job strain model with its three major components of job demands, control, and social support (JDC-S; [44]). It proposes that work environments with high job demands, low job control and autonomy as well as low levels of social support bear the highest risk for adverse health outcomes [43, 44]. With regard to inflammatory processes, studies that draw upon the job strain model are sparse and report inconsistent results [39, 45, 46]. In care professionals, respective investigations based on work stress models are lacking [12, 32].

To this end, we sought to examine associations between psychosocial working conditions based on the JDC-S model and chronic low-grade inflammation among care professionals. As biomarkers of inflammation, CRP as an indicator of humoral immunity and leukocyte count as an indicator of cellular immunity were analyzed [47]: CRP has long been recognized as one of the most sensitive of the acute-phase reactants. It is a key indicator for inflammation in work stress research with potential CRP-upregulation in response to adverse working conditions [12, 19]. Leukocytes are a promising indicator of immune and inflammatory activity for workplace stress research, as leukocyte subpopulation numbers were shown to be altered in individuals under chronic psychosocial stress [12, 48, 49].

Healthcare professionals may represent an at-risk group for disease vulnerability and progression [27]. In particular, geriatric nurses are exposed to various general work stressors such as time pressure, physical demands and interpersonal conflicts, but also specific stressors pertaining to the emotional burden of caring for patients [50, 51]. Exposure to these stressors is linked to burnout, which in turn is not only associated with adverse consequences for general health on an individual level, but also on an organizational level most importantly regarding quality of care and patient safety [27, 52]. Lastly, high rates of absenteeism and intention to leave among nurses are a concern of global scale [53]. It is therefore crucial to understand how working conditions lead to chronic stress with pathophysiological alterations (i.e., low-grade inflammation) and eventually contribute to pathogenesis. A better understanding of those associations may be used for job design and occupational health management. For instance, a recent participatory workplace intervention study was effective in reducing stress-related inflammation among nurses [54, 55].

### Study objectives

Based upon a cross-sectional study, we aimed at exploring individual and synergistic associations of work and individual characteristics with care professionals’ inflammatory outcomes. Specifically, we aimed to determine: (1) the prevalence of increased low-grade inflammation among geriatric care professionals; (2) individual and synergistic associations of risk and protective factors of the work environment with low-grade inflammation outcomes in geriatric care professionals.

## Methods

### Design and ethics

We established a cross-sectional study that combined different data sources of standardized self-reports, medical examinations, and measurement of biomarkers. Our analysis was part of an investigation into care professionals’ age and work environment factors [56]. Prior to the start of the study, ethical approval through the Ethics Committee of the Medical Faculty of Ludwig-Maximilians-University of Munich (No. 99–15) was obtained and agreement was gathered from the study facilities’ management and organization. Before data collection, professionals were informed and provided written consent.

### Sample

Applying a convenience sampling approach, a total of *N* = 140 employees from six geriatric care facilities in South Germany was included in the study. The sample consisted of geriatric care professionals, mainly nurses but also assisting, kitchen, and cleaning staff. Data were collected in 2015 over the course of three months with weekly visits on site. The sample included 111 females (79.3%). 18 (12.9%) care professionals were working part-time and 116 in a shift work schedule (82.9%). Mean age was 44.10 years (standard deviation, *SD* = 12.39, range 18–69 years) with an average professional tenure of 22.32 years (*SD* = 11.98, range 0.5–50 years). Mean weekly working hours were *M* = 37.21 (*SD* = 7.74, range 7–45 hours). Average BMI was 25.63 (*SD* = 4.31, range 18.3–44.0).

### Data collection

The data collection procedure consisted of three consecutive steps: First, a standardized medical history was obtained and an examination was conducted in course of the regular, tri-annual preventive medical check for health care professionals. This assessment is mandatory and performed according to the standards of the German Ordinance on Occupational Health Care [57]. Second, a standardized questionnaire was handed out to each participant. The survey included questions concerning individual characteristics and psychosocial working conditions (all described below). Completed questionnaires were directly returned to the study team in sealed envelopes. Third, a trained occupational physician (study author QC, who also conducted the medical examinations above) withdrew biomarker samples from each professional. Venous blood was collected using serum monovettes (Sarstedt ‘S-Monovette^®^’). Blood samples were immediately stored at 4° Celsius and transferred to the laboratory for further processing. All samples were handled according to standard laboratory procedure. During data collection and further processing, pseudonymization procedures were established through study codes on questionnaires, protocols, and laboratory samples. This allowed matching of survey, examination, and biomarker data. Data were anonymized immediately after data collection.

### Measures and data sources

#### Physician examination

The occupational physician evaluated participants’ current health status and medical history, including acute and chronic diseases with potential relevance to inflammatory reactions (i.e., current infections, tumors, neuroendocrine disorders, rheumatism, arthritis, CVD, recently obtained surgery, or accidents). Information on current medication intake was collected with particular focus on medication affecting inflammatory processes, such as antibiotics, non-steroidal anti-inflammatory drugs, biologicals and cortisone. Further questions included previous GP-provided diagnoses relevant to our study objectives.

#### Professionals’ psychosocial working conditions

Consistent with the job strain model, our questionnaire included three standardized scales for self-evaluation of the nursing work environment that were drawn from a well-established tool for work analysis in healthcare [58]. This tool was developed for healthcare workplaces and has been repeatedly scrutinized for reliability, factorial and content validity [59–61]. We deployed the following scales [59]:

*Work overload* was measured with a three-item scale assessing professionals’ appraisal of quantitative overload and time pressure at work (item example: ‘I often have too much work to do at once’). Answers were obtained on a five-point scale ranging from 1 = ‘no, not at all’ to 5 = ‘yes, to a great extent’. Internal reliability was determined with Cronbach’s α = .88.

*Job autonomy* was assessed with four items (item example: ‘My work allows for decisions on which methods I pursue’). This scale measures skill discretion and degrees of freedom at the workplace (scale range: 1 = ‘no, not at all’ to 5 = ‘yes, to a great extent’). Cronbach’s α was .87.

*Social support* was measured with two questions encompassing key sources of social support at work, i.e., direct supervisor and colleagues; item example: ‘To what extent do you receive social support from your colleagues such that your work is facilitated?’ (scale range: 1 = ‘not at all’ to 4 = ‘to a great extent’). Cronbach’s α was .59.

#### Employment and individual characteristics

The following information on care professionals’ employment and individual (sociodemographic and health) characteristics were gathered to control for potential confounders:

*Employment information* comprised contract (full-time vs. part-time), weekly working hours, and shiftwork (yes vs. no). In addition, employees rated a set of questions concerning adverse work environment conditions (three questions on high noise, poor light, poor climate) as well as physical workload (five items pertaining to demands, e.g., lifting heavy loads, working in unfavorable postures).

*Sociodemographic characteristics* included sex (female, male), age (in years), and professional tenure (in years).

*Health-related information* examined by the physician concerned chronic health conditions and health behaviors including diabetes (no risk vs. risk), risk of CVD (no risk vs. risk), smoking (in pack years), and physical activity in leisure time (yes vs. no). Furthermore, body mass index (BMI) was calculated.

#### Blood samples for biomarkers of inflammation (C-reactive protein, leukocyte count)

*C-reactive protein (CRP)*. Serum concentration of CRP was analyzed by immunoturbidimetric method using the AU600/640/640e/680 and AU2700/5400 Beckman Coulter Analyzers. Laboratory reports listed two categories: values < 5 mg/L were reported non-numeric, values > 5mg/L were reported numerical. Therefore, we used this bivariate outcome classification in our data analyses.

*Leukocyte count*. Leukocytes were analyzed by particle counting (optical-electronic). We considered values between 3.9–10.4 x 10^9^ (women) and 3.9–9.8 x 10^9^ (men), respectively, as normal range based on the reference values provided by our laboratory and following established reference ranges in the respective literature [e.g., 62].

### Statistical analyses

After aggregation of all data sources, prevalence for the outcome variables in the overall sample was determined. Based on the physician’s review of examination and laboratory data, we then identified our study group of interest, i.e., professionals with increased low-grade inflammation (characterized by CRP > 5 mg/L and no further medical conditions or known clinical reason for elevated inflammation).

With regard to the study’s objectives, regression analyses to obtain risk estimates through bivariate (i.e. crude regression estimates) and multivariate analyses (i.e. regression estimates adjusted for all predictor and control variables) were applied. In the main analyses, we included the control variables sex, age, BMI, shiftwork, weekly working time, and in further analyses, additionally CVD risk, diabetes risk, smoking, professional tenure, physical activity in leisure time, adverse environmental conditions, and physical demands. For CRP, we applied binary logistic regressions; for leukocyte counts, we used linear regressions.

Consistent with the propositions of the job strain model, we intended to test two main hypotheses: first, the iso-strain hypothesis suggesting additive, main effects of each component [63]. Second, in line with the buffer hypothesis predicting that protective factors such as autonomy or social support can buffer the potential negative effects of job demands on health and well-being, we tested for statistical interactions between the job demand and resources, respectively [63]. To this end, we explored potential moderation effects by including the interactions of work overload x social support and work overload x autonomy as additional predictors in the multivariate analyses (i.e., identification of multiplicative effects).

Prior to all analyses, continuous predictor variables were standardized (through mean-centering) to limit multi-collinearity. Potential multi-collinearity within the multivariate models was examined using correlation matrices and variance inflation factor [64], and results did not indicate critical collinearity among the predictor variables. As an additional analysis, we also applied the logarithmic transformation to the leukocyte count outcome measure. In order to adjust for multiple testing, we controlled the false discovery rate according to the Benjamini-Hochberg method ($\frac{i}{m}\;\times Q$, where *i* = rank of p-value, *m* = total number of tests and *Q* = false discovery rate) [65]. All analyses were computed with SPSS 26.0 (IBM Inc., Chicago).

## Results

### Overall sample and selection of study group

Altogether, the sample included *N* = 140 care professionals (see S1 Fig in Supporting Information for a flow chart). First, all professionals with an elevated inflammation level due to acute or chronic medical conditions were identified to avoid spurious estimates of associations between predictor and key outcome measures, i.e., chronic low-grade inflammation. After physician’s review, 10 professionals with verified elevated inflammation were excluded (7.1% of the overall group). Medical conditions of excluded professionals were for instance intake of cortisone medication, acute infection, injury from fall, or rheumatism. With regard to sociodemographic and health characteristics, excluded professionals were not significantly different in terms of sex, age, shift work, contract, professional tenure, average working hours, and average BMI compared to the remaining study sample (see S1 Table).

### Descriptive statistics of working conditions and low-grade inflammation

In the study sample (*n* = 130), we identified *n* = 7 professionals (5.4%) with increased low-grade inflammation (CRP > 5 mg/L, yet no further known inflammation-associated medical conditions) and *n* = 123 (94.6%) with no respective indication (CRP < 5 mg/L). Further, we observed a mean leukocyte count of *M* = 6.88 10^9^/L (95% CI [6.66, 7.11]). Both inflammatory endpoints were associated: Leukocyte numbers differed significantly between groups with CRP < 5 mg/L (*M* = 6.81 10^9^/L, 95% CI [6.58, 7.04]) and CRP > 5 mg/L (*M* = 8.20 10^9^/L, 95% CI [7.13, 9.27]; *F*(df = 1) = 7.95, *p* = .006). In Table 1 individual, employment, and psychosocial work characteristics are presented for the total sample (*N* = 140) and both subgroups, respectively.

**Table 1:** Care professionals’ individual, employment, and psychosocial work characteristics (for overall group and subgroups based on CRP cut-off > 5mg/L).

Measures	Overall Group	Subgroups for Analyses		
		CRP > 5mg/L	CRP < 5mg/L	Oneway ANOVA
	*N* = 140	*n* = 7	*n* = 123	
	*M* (*SD*)	*M* (*SD*)	*M* (*SD*)	
*Individual characteristics*				
Age (in years)	44.10 (12.39)	46.43 (7.55)	44.35 (12.52)	*F*(1, 128) = 0.19, *p* = .665
Body mass index	25.63 (4.31)	28.45 (6.24)	25.29 (4.03)	*F*(1, 128) = 3.80, *p* = .053
*Employment characteristics*				
Weekly working time	37.21 (7.74)	40.00 (0.00)	37.12 (7.98)	*F*(1, 126) = 0.78, *p* = .380
*Psychosocial work characteristics*				
Work overload	3.20 (1.06)	3.52 (0.88)	3.12 (1.02)	*F*(1, 128) = 1.03, *p* = .312
Social support	3.06 (0.68)	3.07 (0.79)	3.07 (0.67)	*F*(1, 127) = .00, *p* = .995
Job autonomy	3.23 (1.06)	4.11 (0.67)	3.21 (1.03)	***F*(1, 128) = 5.12, *p* = .025**

*Note*. *n* = 10 (of *N* = 140) participants were excluded from analysis due to acute or chronic inflammation-related medical conditions. *M* = Mean, *SD* = Standard deviation. Significance testing: ANOVA, bold if *p* < .05. Scale ranges of work overload and autonomy: 1 = ‘no, not all’ to 5 = ‘yes, to a great extent’. Scale range of social support scale: 1 = ‘not at all’ to 4 = ‘to a great extent’.

### Associations between working conditions and low-grade inflammation

The results of the regression analyses on bivariate (crude) and multivariate (adjusted) associations between professionals’ individual, employment, and psychosocial work characteristics with inflammatory outcomes (CRP and leukocytes) are depicted in Table 2.

**Table 2:** Crude and adjusted associations of care professionals’ individual, employment, and psychosocial work characteristics with inflammatory markers (C-reactive protein and leukocytes).

		Associations with Outcome							
		C-reactive protein				Leukocytes			
		Crude		Adjusted		Crude		Adjusted	
	Predictors	OR (95% CI)	p-value	OR (95% CI)	p-value	B (95% CI)	p-value	B (95% CI)	p-value
Individual characteristics	Sex (male / female)	1.77 (0.20, 15.31)	.605	1.37 (0.10, 18.12)	.812	0.38 (-0.16, 0.91)	.165	0.52 (-0.09, 1.12)	.093
	Age	1.01 (0.95, 1.08)	.663	1.02 (0.91, 1.14)	.717	0.00 (-0.02, 0.02)	.944	0.00 (-0.02, 0.02)	.899
	Body mass index	1.14 (0.99, 1.32)	.066	1.20 (0.97, 1.48)	.099	-0.01 (-0.06, 0.048)	.825	-0.02 (-0.07, 0.04)	.607
Employment characteristics	Shiftwork (no/yes)	0.39 (0.07, 2.27)	.293	0.59 (0.07, 5.33)	.638	0.02 (-0.57, 0.61)	.951	-0.24 (-0.89, 0.41)	.462
	Weekly working time (in h/w)	1.23 (0.66, 2.31)	.520	1.80 (0.46, 7.10)	.402	0.01 (-0.02, 0.04)	.419	0.02 (-0.01, 0.05)	.210
Psychosocial work characteristics	Work overload	1.55 (0.66, 3.60)	.313	2.29 (0.59, 8.96)	.233	0.08 (-0.15, 0.32)	.491	0.09 (-0.17, 0.35)	.494
	Social support	1.00 (0.46, 2.17)	.995	1.14 (0.37, 3.46)	.823	0.02 (-0.20, 0.25)	.835	0.11 (-0.15, 0.36)	.400
	Autonomy	**3.00 (1.08, 8.39)**	**.036**	**4.10 (1.10, 15.26)**	**.035**	-0.08 (-0.31, 0.15)	.474	-0.11 (-0.35, 0.14)	.402
Model fit		*R*^2^_N_ = 0.00–0.12		*R*^2^_N_ = 0.32		*R*^2^ = 0.00–0.02		*R*^2^ = 0.04	

*Note*. OR = Odds ratio; CI = Confidence interval; B = non-standardized regression coefficient, intercept values not depicted; *R*^2^_N_ = Nagelkerke’s *R*^2^; **bold if *p* < .05**, *n* = 130.

Crude: bivariate regressions (one predictor variable at a time); adjusted: each predictor variable + all other listed variables (sex, age, body mass index, shiftwork, weekly working time, work overload, social support, autonomy)

Concerning the likelihood of elevated CRP levels, the following associations between professionals’ individual and work-related characteristics were observed: There was a weak association between BMI and CRP that was yet neither significant in bi- (crude OR = 1.14, 95% CI [0.99, 1.32], *p* = .066, *R*^2^_N_ = 0.07) nor in multivariate analyses (adjusted OR = 1.20, 95% CI [0.97, 1.48], *p* = .099, *R*^2^_N_ = 0.32). Furthermore, there was a relation between autonomy and CRP such that reports of high autonomy were associated with increased levels of CRP (crude OR = 3.00, 95% CI [1.08, 8.39], *p* = .036, *R*^2^_N_ = *0*.*12*; adjusted OR = 4.10, 95% CI [1.10, 15.26], *p* = .035, *R*^2^_N_ = 0.32). However, following the Benjamini-Hochberg correction with a critical value of $p\left(\frac{1}{8}\;\times .05\right)=0.0625$, it was not statistically significant. We also tested associations with different adjustment sets (i.e., adjusted for age, sex, BMI only and additionally for shiftwork and working time), what yielded similar results (S2 Table).

To test this observed statistical trend, further potential confounding variables were considered. We analyzed the following health-related characteristics and their association with CRP, respectively: CVD risk (no/yes; crude OR = 4.04, 95% CI [0.75, 21.68], *p* = .103), diabetes (no/yes; crude OR = 10.08, 95% CI [0.80, 127.42], *p* = .074), and current and past smoking (in pack years, crude OR = 1.02, 95% CI [0.94, 1.09], *p* = .687). We also deployed professional tenure (instead of age) as a proxy estimate for accumulated exposure to work stressors (crude OR = 1.04, 95% CI [0.97, 1.12], *p* = .250).

Adjustment for these potential confounders in the association between autonomy and CRP did not change the results, i.e., the potential effect of autonomy remained (although not statistically significant after correction for multiple testing). S3 Table reports the adjusted estimates for both outcomes, respectively. Additional control for regular physical activities in leisure time (analogous to Table 2) still showed a possible effect of autonomy on CRP (crude OR = 3.95, 95% CI [0.69, 22.53], *p* = .122, *R*^2^_N_ = 0.07; adjusted OR = 8.09, 95% CI [1.26, 52.02], *p* = .028, *R*^2^_N_ = 0.42).

To rule out potential alternative explanations (e.g., poor physical work environment) and to account for potential contextual influences, we further included professionals’ reports on adverse environmental conditions in the workplace and physical demands in the models. In bivariate analyses, none of the scales was related to CRP levels (adverse environmental conditions: crude OR = 1.20, 95% CI [0.55, 2.62], *p* = .648; physical demands: crude OR = 0.80, 95% CI [0.38, 1.67], *p* = .545). Additional insertion of both scales, respectively, into the multivariate model revealed no significant relationships and did not change the above reported putative effect of job autonomy.

Given the low number of identified participants with increased low-grade inflammation, testing for potential interaction effects between the three job factors and participants’ CRP levels (i.e., JDC-S model’s buffer hypothesis) was undertaken for exploratory reasons only. We neither observed a significant association for the work overload x social support interaction (adjusted OR = 1.18, 95% CI [0.20, 6.79], *p* = .857) nor for the work overload x autonomy interaction (adjusted OR = 0.76, 95% CI [0.14, 4.14], *p* = .753).

Regarding our second outcome leukocyte count, we did not observe significant relationships between the study variables and professionals’ leukocyte numbers neither in the crude nor adjusted models (see Table 2). Adjustment for further health-related variables (CVD risk, diabetes, smoking) did not change these results (see S3 Table). We also applied the logarithmic transformation to the leukocyte count outcome measures, that did not change the results either.

We additionally conducted a sensitivity analysis by repeating the main analyses with the full study sample (*N* = 140; i.e., without exclusion of participants with elevated inflammation due to acute or chronic medical conditions). Concerning CRP, results were similar for individual and employment characteristics, yet there was no longer an association with autonomy, but instead a significant relationship with work overload. For leukocytes, the results were similar to the findings reported above (see S4 Table).

## Discussion

### Findings and potential contributions

Chronic work stress potentially leads to adverse changes in multiple biological systems including the immune system. An emerging research base argues for pathways between exposure to unfavorable workplace conditions and professionals’ chronic systemic low-grade inflammation. However, respective investigations specifically for healthcare professionals are scarce. Drawing upon on an exploratory study into geriatric care professionals’ work factors and inflammatory markers, our preliminary findings contribute to the current knowledge base in various ways.

First, a prevalence of 5.4% of care professionals with critically elevated CRP levels, which were not related to known medical conditions, was determined. Hence, our findings inform future investigations that seek to identify occupational risk groups for disease susceptibility. Follow-up studies are necessary to examine the likelihood of chronic diseases in care professionals with altered inflammatory markers and long-term stress exposure [12, 23, 32].

Second, our study deployed a well-established job-stress model to discern associations between care professionals’ working conditions and inflammatory markers. The application of work stress models rather than aggregate measures and disparate constructs helps to gain a deeper understanding of the fundamental processes of job stress and ensuing dysregulated immune function [12, 45, 66]. Concerning the main propositions of the JDC model, we found no empirical confirmation for the health-impairment process, i.e., deleterious effects of work overload on inflammatory processes. However, we observed a low positive, yet non-significant association between work overload and CRP. Previous JDC-based investigations revealed inconsistent results regarding CRP often with none or weak associations [12, 19, 39, 40, 45, 46]. A meta-analysis on another well-established job-stress model (i.e., effort-reward imbalance, ERI) found an overall, yet small effect of ERI on immunity with stronger effects on mucosal immunity (salivary immunoglobulin A) than on cytokine including CRP as well as leukocyte subsystems [20]. Since the majority of research is based on non-nursing settings, definite inferences concerning the effects of overload on low-grade inflammation in care professionals are premature. Contextual conditions that mitigate the adverse effects of overwork in healthcare should be considered in future research, e.g., opportunities for respite and recovery in care professionals under high work demands [67]. Contrary to our assumptions, we found a tendential positive effect of job autonomy on participants’ CRP levels. This observed trend deserves careful consideration in the light of the current literature and our applied methods. Traditionally, job autonomy has been considered as a fundamental resource for effective task regulation and as beneficial for health and mental well-being [42, 68, 69]. Notwithstanding, high levels of job autonomy have also been associated with poor health and well-being outcomes, also in eldercare professionals [70–73]. One post-hoc explanation for such detrimental effects may be that high job autonomy depletes self-regulatory efforts due to exceeding planning requirements, high demands for self-control, and decreased predictability of work tasks [71]. Our findings thus contribute to investigations into inverse or potentially curvilinear relationships between job autonomy and health outcomes [72, 74]. It has been suggested that physiological dysregulation with sympathetic activation and parasympathetic withdrawal occurs when high autonomy is perceived as an additional stressor [70]. Moreover, whether autonomy functions as a protective factor might depend on individual traits such as self-efficacy [39], in that individuals with low levels may perceive a high degree of autonomy as overcharging and hence show stress reactions. Future investigations should thus scrutinize potential harmful effects of job autonomy on immunological processes.

Third, the associations were not uniform for both study outcomes. CRP is one of the most frequently studied inflammatory markers and is suggested to be positively associated with work stress [12]. In contrast, the study base on leukocytes is limited so far and findings show diverse or no associations with workplace stress, especially when assessed with the JDC(-S) model [12, 19]. Leukocytes as a marker of cellular immunity are expected to decrease in number in response to chronic stress [12]. Effects of workplace-related stress on leukocyte levels in particular need to be further investigated; in general, consideration of several molecular-biological (i.e., humoral, cellular, and intracellular) levels of the immune system in work stress research would be desirable and allow to discern potential confluent or disparate effects of job factors on different branches of the immune system.

We explored potential multiplicative effects according to the buffer hypothesis of the JDC-S model [63]. Despite the constraints of this survey (i.e., limited prevalence of low-grade inflammation), we tested for moderating effects to obtain further insights into possible interdependencies between the job factors in nursing. Our null findings are consistent with reviews suggesting that moderating influences of control and social support lack substantial empirical confirmation [63, 75, 76].

### Limitations

Our observations should be interpreted in the light of important limitations. Firstly, we used a cross-sectional design what limits inferences on causality and long-term effects. In cross-sectional studies, the “level of chronicity” [12] of stress experience is often not considered, yet inflammation-related processes are suggested to vary in different stages of stress [77]. Although we have included important proxy variables such as professional tenure, the duration of job stress may not have been comparable in our sample with possible effects on the biomarkers. Future studies should consider the level of chronicity by collating multiple information including psychological symptoms, in order to differentiate between individuals in different phases of job stress—from acute stages up to burnout [9, 12]. Moreover, peripheral inflammation is modulated by other human stress systems through complex neuroendocrine-immune cascades and interactions, and should thus not be investigated in isolation in future research, but in its interplay with other stress markers, in particular the hypothalamus-pituitary adrenal axis hormone cortisol [78].

Our data stemmed from geriatric care professionals, who underwent a standardized, periodic health examination. This may limit generalizability to other nursing work environments. Yet, regularly scheduled examinations reduce the probability of self-selection bias. We acknowledge the unequal distribution of men and women what limits inferences concerning potential sex differences in (work) stress-related inflammatory responses [16, 41].

Furthermore, we are aware that for CRP different methods and cut-offs are used depending on the research subject or clinical indication. In our study, a cut-off of 5 mg/L was applied following our study’s laboratory standard reporting procedure. In another study based on a healthy working sample a similar cut-off was deployed [79]. Yet, in several other studies, high-sensitivity CRP was used with lower detection limits [16, 18, 39], providing greater resolution in lower CRP-concentrations. In this regard, it is also noteworthy that we were restrictive in excluding participants with elevated CRP concentrations due to known medical reasons for inflammation. Future research should elaborate consistent methods for instance by defining clear thresholds and exclusion criteria for better comparability and replicability of findings. The importance of this aspect was reflected in the results of our additional sensitivity analysis, which were different for CRP, when no exclusion criteria were applied. Indeed, it is crucial to thoroughly distinguish chronic systemic low-grade inflammation from reactions to infection or injury, in order to capture inflammation induced by *psychological* stress rather than by medical conditions [9, 80].

Moreover, although we controlled for a broad set of confounders, we acknowledge that further factors outside the work environment may influence the interplay of occupational conditions and immunological processes. Future investigations should strive to control for potentially amplifying but also protective functions of individual behaviors, personal characteristics, and social circumstances. For example, job strain in combination with caregiving to a relative was shown to have the strongest adverse effects on physiological functioning in civil servants [81]. On the other hand, regular and efficient sleep may mitigate inflammatory processes in nursing professionals [82]. Moreover, specific personal resources might be protective against adverse effects of work stress on immune function, as was shown for trait mindfulness among care workers [83]. Another limitation in this context is that we did not control for participants’ respective profession due to confidentiality measures.

Finally, this exploratory study was based on a convenience sample with a limited number of participants. In the original study, we tried to recruit as many participants as possible through various measures. However, like in other applied biomarker studies, we faced the challenge of large exclusion rates of participants because of appointment cancellations (e.g., due to spontaneous shift changes, sick leave, holiday), medical reasons (e.g., specific medical or psychiatric conditions affecting blood levels), personal reasons (e.g., refusal to provide sample) or other reasons such as pregnancy. In view of the low prevalence of the outcome, our study was strongly underpowered and therefore, results can only be interpreted with great caution. Future studies ought to replicate observed effects with larger samples to achieve greater power. In addition, the results regarding social support in particular should be cautiously considered, as this measure showed low internal consistency, perhaps due to the limited number of merely two items.

### Implications for research and nursing practice

Regarding further research, this study advocates the viability of inflammatory markers in the quest for work-related influences on care professionals’ health. Yet, prospective studies on accumulated exposures to adverse working conditions and immunological status over time are necessary [19, 27]. Ensuing research should also consider the utility of other indicators of inflammation, e.g., cytokine imbalance as a composite measure of pro-inflammatory and anti-inflammatory expression [12]. Moreover, the application of a holistic approach, as per the allostatic load index, a multi-system indicator of wear-and tear effects on brain and body, may give deeper insights into how chronic work stress in nursing leads to different adverse health outcomes in the long-term [84–87]. Consequently, this may contribute to attempts to quantify current or future disease risks in nursing samples, for example with identification of immune-risk phenotypes [32]. Future studies should also examine the consistency of our observations by applying alternative job-stress models (such as ERI) and by including other job stressors, such as those specific to healthcare (i.e., caring for suffering patients), or organizational stressors, like job insecurity and experience of injustice [16, 27, 66]. Besides the traditional work stressors, also other, more severe stressors, such as workplace bullying or harassment, should be subjected to future research. Those kinds of stressors may be perceived as threatening to psychosocial safety and may elicit severe stress reactions in individuals [88]. Thus, the choice of exposure measures might affect the potential to capture work stress at a physiologically detectable level.

Concerning implications for nursing practice, our results underline the need for further measures to promote nurses’ well-being and health. Given their high workload and intense demands, care professionals may constitute a vulnerable population. Since low-grade inflammation is suggested as a powerful predictor of chronic diseases [9–11], inflammatory markers may represent an important leverage point for identification of health status and potential need for action. For one thing, monitoring of inflammatory markers as indicators of dysregulated stress-physiological functioning in the course of standard medical examinations could help to identify at-risk professionals for detrimental health outcomes. For another thing, workplace interventions could be implemented to improve inflammatory processes: in healthcare professionals, meditation- and mindfulness-based trainings were shown to alter pro-inflammatory gene expression [89, 90]; across different occupational settings, physical activity interventions were demonstrated to decrease employees’ CRP levels [19].

## Conclusion

Chronic low-grade inflammation has become increasingly important for our understanding of the pathogenesis of (work) stress-related diseases. Taken together, this exploratory study provides valuable insights into potential biological correlates of psychosocial work stress in care professionals. Given the study’s limitations, the findings are preliminary and their interpretation warrant caution. Further research is needed to clarify the role of job demands as well as resources for immune function in healthcare workers.

## Supporting information

S1 Fig Flow chart describing step-wise selection of study sample. (TIF)

S1 Table Comparison of excluded participants with study sample regarding sociodemographic and employment-related information. (DOCX)

S2 Table Crude and adjusted associations (including different adjustment sets, Model 1–3) of care professionals’ individual, employment, and psychosocial work characteristics with C-reactive protein. (DOCX)

S3 Table Expanded list of care professionals’ individual, employment, and psychosocial work characteristics and associations with inflammatory markers (C-reactive protein and leukocytes). (DOCX)

S4 Table Crude and adjusted associations of care professionals’ individual, employment, and psychosocial work characteristics with inflammatory markers (C-reactive protein and leukocytes), full sample. (DOCX)
