# Supplementary figures and images for: Psychosocial working conditions and chronic low-grade inflammation in geriatric care professionals: A cross-sectional study
Source: PLoS One. 2022 Sep 15;17(9):e0274202. doi: 10.1371/journal.pone.0274202 (PMC9477283; doi:10.1371/journal.pone.0274202)

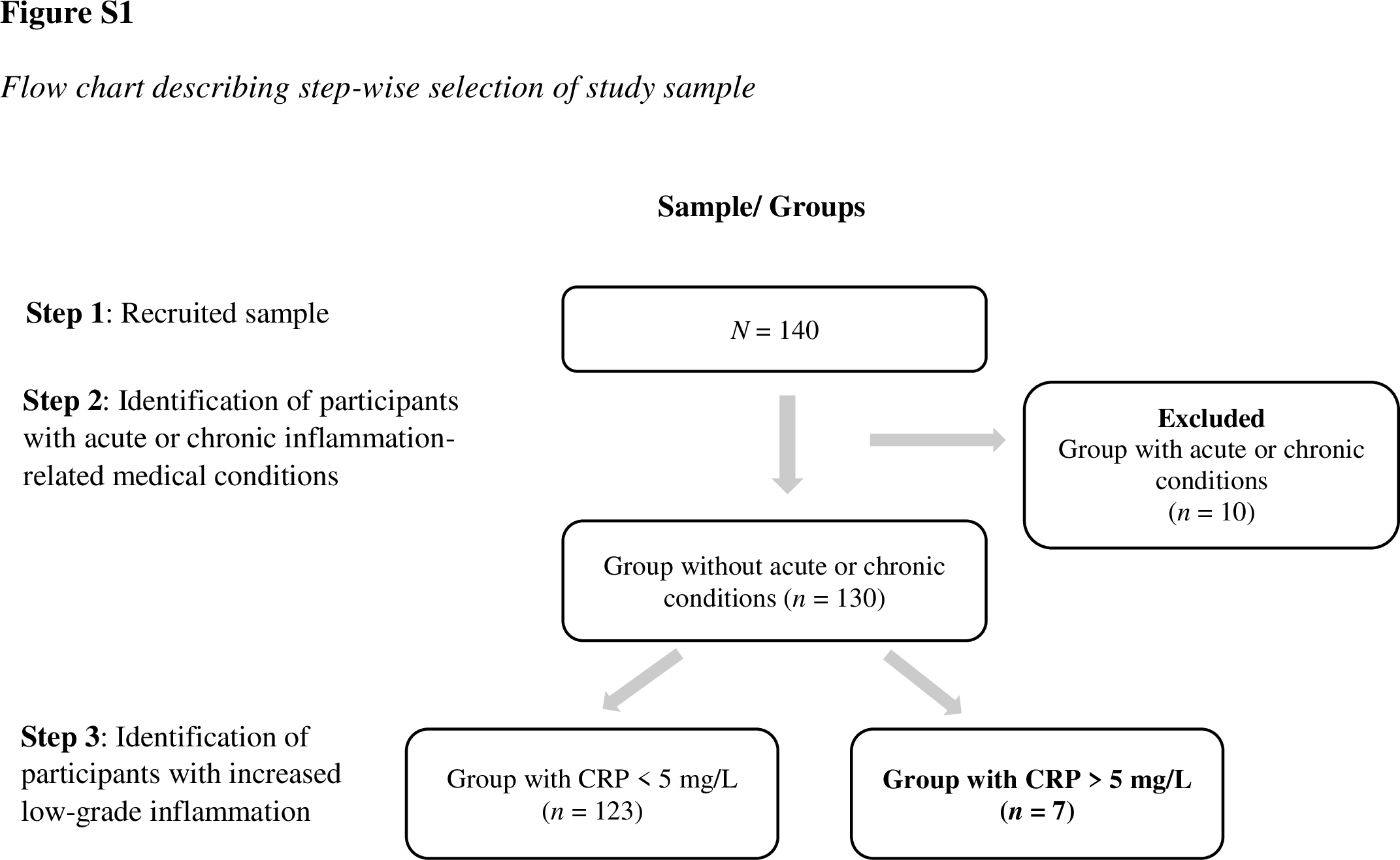

Supplement: S1 Fig — (TIF) [file pone.0274202.s001.tif]
