# Supplementary material for: Psychosocial working conditions and chronic low-grade inflammation in geriatric care professionals: A cross-sectional study
Source: PLoS One. 2022 Sep 15;17(9):e0274202. doi: 10.1371/journal.pone.0274202 (PMC9477283; doi:10.1371/journal.pone.0274202)
Supplement: S1 Table — (DOCX) [file pone.0274202.s002.docx]

**Table S1**

*Comparison of excluded* *participants with study sample regarding sociodemographic and employment-related information.*

| Measures | | **Study Sample** | **Excluded participants with elevated inflammation due to acute or chronic medical conditions** |  |
| --- | --- | --- | --- | --- |
|  |  | *n* = 130 | *n* = 10 | **Chi-Square-Test/ One-way ANOVA** |
|  | | Frequencies (*n*)/ *M (SD)* | |  |
| *Sociodemographic characteristics* | |  | |  |
|  | Sex | 101 females | 10 females | Chi^2^(df = 1) = 2.81, *p* = .093 |
|  | Age (in years) | 44.46 (12.29) | 39.40 (13.33) | *F*(df = 1) = 1.56, *p* = .214 |
|  | Body mass index | 25.46 (4.20) | 27.75 (5.27) | *F*(df = 1) = 2.64, *p* = .106 |
| *Employment characteristics* | |  |  |  |
|  | Shiftwork (yes) | 107 | 9 | Chi^2^(df = 1) = .33, *p* = .563 |
| Contract (full-time) | | 111 | 7 | Chi^2^(df = 1) = 2.64, *p* = .104 |
|  | Weekly working time | 37.26 (7.81) | 36.50 (7.04) | *F*(df = 1) = .08, *p* = .778 |
|  | Professional tenure | 22.70 (12.04) | 17.30 (10.41) | *F*(df = 1) = 1.90, *p* = .170 |

*Note. N* = 140
