# Supplementary material for: Psychosocial working conditions and chronic low-grade inflammation in geriatric care professionals: A cross-sectional study
Source: PLoS One. 2022 Sep 15;17(9):e0274202. doi: 10.1371/journal.pone.0274202 (PMC9477283; doi:10.1371/journal.pone.0274202)
Supplement: S2 Table — (DOCX) [file pone.0274202.s003.docx]

**Table S2**

*Crude and adjusted associations (including different adjustment sets, Model 1-3) of care professionals’ individual, employment, and psychosocial work characteristics with C-reactive protein*

|  |  | **Associations with Outcome: C-reactive protein** | | | |  |  |  |  |
| --- | --- | --- | --- | --- | --- | --- | --- | --- | --- |
|  |  | Crude |  | Model 1 |  | Model 2 |  | Model 3 |  |
|  | Predictors | OR (95% CI) | p-value | OR (95% CI) | p-value | B (95% CI) | p-value | OR (95% CI) | p-value |
| Individual characteristics | Sex (male / female) | 1.77 (0.20, 15.31) | .605 | - |  | - |  | 1.37 (0.10, 18.12) | .812 |
|  | Age | 1.01 (0.95, 1.08) | .663 | - |  | - |  | 1.02 (0.91, 1.14) | .717 |
|  | Body mass index | 1.14 (0.99, 1.32) | .066 | - |  | - |  | 1.20 (0.97, 1.48) | .099 |
| Employment characteristics | Shiftwork (no/yes) | 0.39 (0.07, 2.27) | .293 | - |  | - |  | 0.59 (0.07, 5.33) | .638 |
|  | Weekly working time (in h/w) | 1.23 (0.66, 2.31) | .520 | - |  | - |  | 1.80 (0.46, 7.10) | .402 |
| Psychosocial work characteristics | Work overload | 1.55 (0.66, 3.60) | .313 | 1.31 (0.53, 3.25) | .556 | 1.82 (0.56, 5.89) | .318 | 2.29 (0.59, 8.96) | .233 |
|  | Social support | 1.00 (0.46, 2.17) | .995 | 1.23 (0.54, 2.81) | .631 | 1.01 (0.40, 2.56) | .977 | 1.14 (0.37, 3.46) | .823 |
|  | Autonomy | **3.00 (1.08, 8.39)** | **.036** | **3.36 (1.18, 9.53)** | **.023** | **3.78 (1.06, 13.40)** | **.040** | **4.10 (1.10, 15.26)** | **.035** |
| Model fit | | *R*^2^_N_ = 0.00 – 0.12 | | *R*^2^ = 0.09 – 0.22 | | *R*^2^ = 0.16 - 0.28 | | *R*^2^_N_ = 0.32 | |

*Note.* OR = Odds ratio; CI = Confidence interval; *R*^2^_N_ = Nagelkerke’s *R*^2^; **bold if p < .05**, *n* = 130**.**

**Crude**: bivariate regressions (one predictor variable at a time);

**Model 1**: Multivariate regressions adjusted for sex, age, body mass index

**Model 2**: Multivariate regressions adjusted for sex, age, body mass index + shiftwork, weekly working time

**Model 3**: Multivariate regressions, fully adjusted (adjusted for sex, age, body mass index + shiftwork, weekly working time + social support, autonomy/ work overload, autonomy/ work overload, social support)
