# Supplementary material for: Psychosocial working conditions and chronic low-grade inflammation in geriatric care professionals: A cross-sectional study
Source: PLoS One. 2022 Sep 15;17(9):e0274202. doi: 10.1371/journal.pone.0274202 (PMC9477283; doi:10.1371/journal.pone.0274202)
Supplement: S3 Table — (DOCX) [file pone.0274202.s004.docx]

**Table S3**

|  |  | **Associations with Outcome:** | | | |  | |
| --- | --- | --- | --- | --- | --- | --- | --- |
|  |  | **C-reactive protein** |  |  | **Leukocytes** |  | |
|  |  | OR (95% CI) | p-value |  | B (95% CI) | | p-value |
| Predictors |  | adjusted |  |  | adjusted | |  |
| Individual characteristics | Sex (male / female) | 0.80 (0.04, 14.94) | .878 |  | 0.53 (-0.09, 1.15) | | .093 |
|  | Age | 1.00 (0.88, 1.14) | .982 |  | 0.00 (-0.02, 0.02) | | .899 |
|  | Body mass index | 1.10 (0.83, 1.46) | .494 |  | -0.02 (-0.08, 0.05) | | .650 |
|  | CVD risk (no/yes) | 4.18 (0.20, 85.86) | .354 |  | 0.02 (-0.63, 0.67) | | .948 |
|  | Diabetes (no/yes) | 16.59 (0.45, 610.75) | .127 |  | -0.47 (-2.36, 1.42) | | .622 |
|  | Nicotine use (in pack years) | 0.92 (0.79, 1.07) | .270 |  | 0.00 (-0.03, 0.03) | | .831 |
| Employment characteristics | Shiftwork (no/yes) | 0.63 (0.05, 7.37) | .714 |  | -0.25 (-0.92, 0.42) | | .462 |
|  | Weekly working time (in h/w) | 1.65 (0.41, 6.59) | .480 |  | 0.02 (-0.01, 0.05) | | .236 |
| Psychosocial work characteristics | Work overload | 2.92 (0.60, 14.17) | .185 |  | 0.09 (-0.17, 0.35) | | .491 |
|  | Social support | 1.10 (0.34, 3.59) | .873 |  | 0.10 (-0.16, 0.36) | | .451 |
|  | Autonomy | **4.87 (1.05, 22.64)** | **.043** |  | -0.10 (-0.36, 0.17) | | .471 |

*Expanded list of care professionals’ individual, employment, and psychosocial work characteristics and associations with inflammatory markers (C-reactive protein and leukocytes)*

*Note*. OR = Odds ratio; 95% CI = Confidence interval; B = non-standardized regression coefficient, intercept values not depicted; adjusted for all listed variables; **bold if p < .05,** *n* = 130.
