# Supplementary material for: Psychosocial working conditions and chronic low-grade inflammation in geriatric care professionals: A cross-sectional study
Source: PLoS One. 2022 Sep 15;17(9):e0274202. doi: 10.1371/journal.pone.0274202 (PMC9477283; doi:10.1371/journal.pone.0274202)
Supplement: S4 Table — (DOCX) [file pone.0274202.s005.docx]

|  |  | **Associations with Outcome** | | | | | | | | |
| --- | --- | --- | --- | --- | --- | --- | --- | --- | --- | --- |
|  |  | **C-reactive protein** | |  |  |  | **Leukocytes** |  |  |  |
|  |  | Crude |  | Adjusted |  |  | Crude |  | Adjusted |  |
|  | Predictors | OR (95% CI) | p-value | OR (95% CI) | p-value |  | B (95% CI) | p-value | B (95% CI) | p-value |
| Individual characteristics | Sex (male / female) | 4.72 (0.60, 37.14) | .141 | 4.51 (0.49, 41.56) | .184 |  | 0.52 (-0.10, 1.14) | .101 | **0.70 (0.02, 1.38)** | **.043** |
|  | Age | 0.99 (0.95, 1.03) | .521 | 0.98 (0.93, 1.03) | .343 |  | 0.00 (-0.03, 0.02) | .594 | -0.01 (-0.03, 0.01) | .527 |
|  | Body mass index | **1.14 (1.02, 1.26)** | **.019** | 1.09 (0.96, 1.23) | .176 |  | 0.05 (-0.01, 0.11) | .114 | 0.02 (-0.04, 0.08) | .532 |
| Employment characteristics | Shiftwork (no/yes) | 0.84 (0.22, 3.23) | .801 | 0.56 (0.11, 2.94) | .494 |  | 0.26 (-0.42, 0.94) | .452 | -0.14 (-0.86, 0.58) | .700 |
|  | Weekly working time (in h/w) | 1.02 (0.94, 1.10) | .714 | 1.00 (0.91, 1.11) | .941 |  | 0.02 (-0.02, 0.05) | .294 | 0.02 (-0.02, 0.06) | .279 |
| Psychosocial work characteristics | Work overload | **1.84 (1.04, 3.23)** | **.035** | **2.33 (1.15, 4.70)** | **.019** |  | 0.09 (-0.16, 0.35) | .464 | 0.16 (-0.11, 0.43) | .239 |
|  | Social support | 0.90 (0.54, 1.50) | .690 | 1.01 (0.52, 1.97) | .984 |  | 0.05 (-0.20, 0.31) | .685 | 0.14 (-0.14, 0.41) | .332 |
|  | Autonomy | 1.12 (0.67, 1.88) | .655 | 1.19 (0.66, 2.14) | .564 |  | -0.12 (-0.37, 0.13) | .350 | -0.11 (-0.38, 0.15) | .393 |
| Model fit | | *R*^2^_N_ = 0.00 – 0.07 | | *R*^2^_N_ = 0.21 | |  | *R*^2^ = 0.00 – 0.02 | | *R*^2^ = 0.07 | |

**Table S4**

*Crude and adjusted associations of care professionals’ individual, employment, and psychosocial work characteristics with inflammatory markers (C-reactive protein and leukocytes), full sample*

*Note*. OR = Odds ratio; CI = Confidence interval; B = non-standardized regression coefficient, intercept values not depicted; *R*^2^_N_ = Nagelkerke’s *R*^2^; **bold if *p* < .05**, *n* = 140.

Crude: bivariate regressions (one predictor variable at a time); adjusted: each predictor variable + all other listed variables (sex, age, body mass index, shiftwork, weekly working time, work overload, social support, autonomy)
